# Common and Uncommon CT Findings in CVID-Related GL-ILD: Correlations with Clinical Parameters, Therapeutic Decisions and Potential Implications in the Differential Diagnosis

**DOI:** 10.1007/s10875-023-01552-1

**Published:** 2023-08-07

**Authors:** Riccardo Scarpa, Francesco Cinetto, Cinzia Milito, Sabrina Gianese, Valentina Soccodato, Helena Buso, Giulia Garzi, Maria Carrabba, Emanuele Messina, Valeria Panebianco, Carlo Catalano, Giovanni Morana, Vassilios Lougaris, Nicholas Landini, Maria Pia Bondioni

**Affiliations:** 1 Department of Medicine, DIMED, University of Padova, Padova, Italy; 2 Internal Medicine 1, Ca’ Foncello University Hospital, AULSS2, Treviso, Italy; 3 Department of Molecular Medicine, “Sapienza” University of Rome, Rome, Italy; 4 Internal Medicine Department, Rare Disease Unit, Fondazione IRCCS Ca’ Granda Ospedale Maggiore Policlinico, Milan, Italy; 5 Department of Radiological Sciences, Oncology and Pathology, Policlinico Umberto I, “Sapienza” University, Rome, Italy; 6 Department of Radiology, Ca’ Foncello General Hospital, Treviso, Italy; 7 Department of Clinical and Experimental Sciences, Pediatrics Clinic and Institute for Molecular Medicine A. Nocivelli, University of Brescia, Brescia, Italy; 8 ASST-Spedali Civili di Brescia, Brescia, Italy; 9 Radiology Unit, ASST-Spedali Civili di Brescia, Brescia, Italy

**Keywords:** Chest CT, CVID, GL-ILD, airway disease, interstitial lung disease, bronchiectasis

## Abstract

**Purpose:**

To investigate computed tomography (CT) findings of Granulomatous Lymphocytic Interstitial Lung Disease (GL-ILD) in Common Variable Immunodeficiency (CVID), also in comparison with non-GL-ILD abnormalities, correlating GL-ILD features with functional/immunological parameters and looking for GL-ILD therapy predictive elements.

**Methods:**

CT features of 38 GL-ILD and 38 matched non-GL-ILD subjects were retrospectively described. Correlations of GL-ILD features with functional/immunological features were assessed. A logistic regression was performed to find a predictive model of GL-ILD therapeutic decisions.

**Results:**

Most common GL-ILD CT findings were bronchiectasis, non-perilymphatic nodules, consolidations, Ground Glass Opacities (GGO), bands and enlarged lymphnodes. GL-ILD was usually predominant in lower fields. Multiple small nodules (≤10 mm), consolidations, reticulations and fibrotic ILD are more indicative of GL-ILD. Bronchiectasis, GGO, Reticulations and fibrotic ILD correlated with decreased lung performance. Bronchiectasis, GGO and fibrotic ILD were associated with low IgA levels, whereas high CD4+ T cells percentage was related to GGO. Twenty out of 38 patients underwent GL-ILD therapy. A model combining Marginal Zone (MZ) B cells percentage, IgA levels, lower field consolidations and lymphnodes enlargement showed a good discriminatory capacity with regards to GL-ILD treatment.

**Conclusions:**

GL-ILD is a lower field predominant disease, commonly characterized by bronchiectasis, non-perilymphatic small nodules, consolidations, GGO and bands. Multiple small nodules, consolidations, reticulations and fibrotic ILD may suggest the presence of GL-ILD in CVID. MZ B cells percentage, IgA levels at diagnosis, lower field consolidations and mediastinal lymphnodes enlargement may predict the need of a specific GL-ILD therapy.

**Supplementary Information:**

The online version contains supplementary material available at 10.1007/s10875-023-01552-1.

## Introduction

Common Variable Immunodeficiency (CVID) [1] is a primary immunodeficiency characterized by decreased IgG, IgA and/or IgM serum levels and impaired antibody response to immunization or infections [2]. CVID results in a broad spectrum of clinical presentations, including infectious [3] and non-infectious complications, as chronic airways abnormalities and immune-mediated interstitial lung disease (ILD) [4].

Possible manifestations of ILD are follicular bronchiolitis, nodular lymphoid hyperplasia, granulomatous lung disease, lymphocytic interstitial pneumonia, non-specific interstitial pneumonia and organizing pneumonia [1].

Since all these patterns may be present in the same patient, the term “Granulomatous and Lymphocytic Interstitial Lung Disease (GL-ILD)” was coined for CVID, referring to “a distinct clinico-radio-pathological ILD, associated with a lymphocytic infiltrate and/or granuloma in the lung” [5]. GL-ILD is a rare manifestation of a rare disease [6], identified in around 8–20% of CVID subjects, resulting from a systemic immune dysregulation. This is underlined by the association with other immune-mediated complications and by the alteration in T and B lymphocytes compartment, such as lower percentage of switched-memory and marginal zone (MZ) B cells, increased percentage of circulating CD21 low B cells and a preferential memory CD4+ T cell differentiation toward a CXCR3+CCR6- TH1 phenotype [7, 8]. Previous studies suggested that GL-ILD patients are at higher risk of B-cell lymphoma, too [9]. According to the UK-PID Network Consensus [5], in the suspicion of GL-ILD, it is useful in performing chest computed tomography (CT) examination, lung function tests, bronchoscopy and surgical lung biopsy, that is mandatory for a definite diagnosis, but not free from risks [10]. The main CT GL-ILD features are nodules, ground glass opacities, reticulations, consolidations, and interstitial fibrosis [11]. Moreover, CT may evaluate chronic bronchial abnormalities, such as bronchiectasis [1]. Since GL-ILD patients may be asymptomatic or have non-specific symptoms, CT may identify suspicious features in advance, anticipating clinical and pulmonary function tests (PFT) abnormalities [10] and providing prognostic elements [12]. Hence, some GL-ILD CT scores have been proposed to evaluate lung involvement [13–15], although there is not a general consensus, yet. Furthermore, the radiological differential diagnosis with other ILD and between acute and chronic lung manifestations may be challenging [5]. In particular, sarcoidosis is considered in differential diagnosis [5, 16], while, on a background of GL-ILD abnormalities, the superimposition of acute infections may be hard to identify, as well as lung lymphoma [5]. Lastly, there is no evidence of any specific radiological element that may be associated with the need of GL-ILD therapy.

Thus, the primary aim of this work was to describe common and uncommon CT findings in a cohort of CVID patients with GL-ILD, in comparison with non-GL-ILD patients and also from the perspective of a differential diagnosis, in order to provide elements for a confident radiologic diagnosis. We also tested the correlations with functional and immunological features and sought any relevant element (radiological and non-radiological) associated with consequent GL-ILD therapeutic decisions.

## Material and Methods

### Study Design

This was a multicentric retrospective study involving four Referral Care Centers for Primary Immunodeficiencies (Rome, Padua, Milan and Brescia). The electronic archives of each center were searched to identify CVID patients with GL-ILD, from 2018 to 2021, looking for the first available chest CT raising the suspicion of GL-ILD.

Inclusion criteria were CVID diagnosed according to the ESID criteria (http://esid.org/Working-Parties/Registry/Diagnosis-criteria), available chest CT examination performed before any GL-ILD treatment, confirmed or suspected GL-ILD diagnosis according to UK-PID Network Consensus. In particular, a confirmed diagnosis required the presence of radiological abnormalities consistent with GL-ILD, with or without signs of lung functional impairment, especially of gas transfer and, presence of typical histopathological evidences as granulomatous inflammation, peribronchiolar lymphoid proliferation, interstitial lymphoid proliferation, and CD4-cell predominance [5]. GLILD was defined as suspected in the presence of all the above apart from histopathological evidence [5]. All patients with suspected GL-ILD also had to present a GL-ILD probability score >50% to be included [9].

Exclusion criteria were clinical suspicion of a pulmonary infectious disease or lymphoma, GL-ILD in active or previous therapy, refusal to sign the informed consent.

We also searched for age- and sex–matched CVID non-GL-ILD patients to perform the radiological comparison, following the same other inclusion and exclusion criteria.

Demographic, clinical, laboratory data and PFT at time of CT scan were recorded for all patients, as well as the presence of granulomas, in available biopsy specimens. Moreover, data on IgG replacement therapy, the need for antibiotic prophylaxis and any subsequent GL-ILD specific treatment were noted.

This study was approved by the Ethics Committee of “Sapienza” University of Rome (CE 4694/, n. 316/2016) and was performed in accordance with the Good Clinical Practice guidelines, the International Conference on Harmonization guidelines and the Declaration of Helsinki.

### Imaging Analysis

Two thoracic radiologists of 7 and 20 years of experience (NL and MPB) reviewed the literature to define a score that could describe CT findings of both GL-ILD and lung diseases usually considered in differential diagnosis [11–22].

Thus, the following CT features were assessed for airways and parenchyma: presence of bronchiectasis, bronchial wall thickening (internal diameter of airway lumen less than 80% of its external diameter or bronchial wall at least twice as thick as that of normal airways [12]), mucous plugs, tree in bud, mosaic perfusion, nodules, consolidation, ground glass opacities (GGO), reticulations, fibrotic ILD (defined as consolidation, GGO, reticulations with architectural distortions and bronchiectasis, as well as honeycombing), cavitation/necrosis, parenchymal scars/bands. The severity of bronchiectasis was also scored, based on the highest observed ratio between bronchial lumen and vessel diameter (mild=1–1.5 vessel diameter; medium=1.5–2.0; high>2.0 [13]), as well as their prevalent location (central, peripheral, diffuse). Nodules were divided in small and large, based on their dimension (≤10 mm or >10 mm [22]), defining the amount (≤3 or >3, namely multiple), the density (solid, ground glass, both solid nodules and ground glass nodules), the presence of halo sign around solid nodules or inner calcifications. Small nodules preferential distribution (perilymphatic, centrilobular, random) was also recorded. Large nodules shape (round/oval, lobulated, irregular) and margins (sharp or ill defined) were assessed. All CT findings were defined according to the glossary terms of the Fleishner society [23] or, when a definition was not available, by referring to literature. Peripheral areas were defined as 2–3 cm from the pleura [24]. The score was also adopted to describe alterations independently in upper and lower fields, adopting the carina as landmark [25], and assessing if CT abnormalities were present in one of the two fields only. Then, the whole disease was assessed as predominant in upper, lower fields or without predominance. Additional findings were presence of pleural effusion, enlarged mediastinal lymph nodes (brevis axis >1 cm), calcified enlarged lymph nodes, pericardial effusion. The readers assessed the score independently, resolving disagreements by consensus. Lung alterations were assessed with a standard lung or soft tissue window, depending on the finding to be assessed. Multiplanar reconstructions could be utilized.

### Statistical Analysis

As descriptive statistics, we reported absolute count and percentage of cases for qualitative data and median with interquartile range (IQR) for quantitative data. Chi-squared and Fisher’s exact tests were used to assess significance for categorical variables. For continuous variables, Shapiro-Wilk test and quantile-quantile plots inspection were used to assess normality of distributions. Significance was verified using a proper *t*-test and a two-tailed *p*-value below 0.05 was considered statistically significant. Univariate and multivariable logistic regression models were fitted to calculate odds ratios (OR), 95% confidence intervals (CI) and the area under the curve (AUC) of receiver operating characteristic (ROC) curves. Variables entered in the multivariate model were chosen based either on clinical data and existing literature and on data-driven variable selection methods to optimize model’ robustness [26]. Statistical analyses were performed with Prism, release 9.4.0 (© 2022 GraphPad Software).

## Results

### Patient Characteristics

The search identified 38 patients (15 male), median age 44.5 years and median disease duration 9 years. Twenty-eight patients were never smokers, 3 were former and 7 current smokers. Bioptic sampling for a definite diagnosis was performed in 30/38 patients, 18 of them presenting histological evidence of granuloma.

All patients were on IgG replacement treatment with a median trough level of 833 mg/dl. Eighteen patients were also taking antibiotic prophylaxis and 20 (53%) patients underwent GL-ILD specific treatment after the CT. Overall, 13 patients received glucocorticoids: 5 as monotherapy and 8 in combination with immunosuppressant. Anti-CD20 monoclonal antibodies were administered in 10 patients, mycophenolate mofetil in 5 and azathioprine in 1.

The majority of GL-ILD patients had splenomegaly on ultrasound examination (92%) and 21 patients (55%) had a history of autoimmune cytopenia with a prevalence of immune thrombocytopenic purpura. B cells subpopulation analysis, according to EUROclass [27] identified a median percentage of CD21 low B cells of 13.9.

Thirty-eight age- and sex-matched CVID non-GLILD patients were enrolled as control. All demographics, clinical and laboratory characteristics and data on PFT are summarized in Table 1, respectively.

**Table 1:** Patients’ characteristics: demographics, clinical, laboratory data and pulmonary function tests

a)				
Characteristics	All CVID	GL-ILD	NON GL-ILD	*p*-value
Patients, *n* (%)	76 (100)	38 (100)	38 (100)	
Females, *n* (%)	47 (62)	23 (61)	24 (63)	ns
Age (yrs), median (IQR)	50 (40–61)	50 (40–64)	51 (39–60)	ns
Age (yrs), median (IQR) at CVID diagnosis	36 (24–44.5)	36 (24–44)	36 (24–46)	ns
Disease duration (yrs)*, median (IQR)	8 (4–17)	10 (4–20)	7 (4–15)	ns
Smoking status, *n* (%)				
• never	60 (79)	28 (74)	32 (84)	
• former	6 (8)	3 (8)	3 (8)	
• current	10 (13)	7 (18)	3 (8)	
Splenomegaly, *n* (%)	51 (67)	35 (92)	16 (42)	<0.001
Autoimmune cytopenia, *n* (%)	25 (33)	21 (55)	4 (11)	0.001
ITP, *n* (%)	26 (34)	20 (53)	6 (16)	0.003
SmB (%), median (IQR)	1.8 (1–5.4)	1.5 (0.5–3.9)	4.1 (1–7.1)	ns
CD21low (%), median (IQR)	13 (6–30)	13.9 (5.8–30.2)	8.2 (5.5–21.1)	ns
MZ (%), median (IQR)	8 (2–19)	5.8 (1.4–12.1)	14.1 (8.5–18.2)	0.011
IgA at diagnosis (mg/dl), median (IQR)	6 (1–22)	5 (1–19)	8.5 (0.5–28)	ns
IgG at diagnosis (mg/dl), median (IQR)	236 (86–371)	238.5 (62–355)	186 (100–423.5)	ns
IgM at diagnosis (mg/dl), median (IQR)	18 (3–34)	12 (3–30)	19 (4.6–46)	ns
IgG TL (mg/dl), median (IQR)	752 (632–870.8)	833 (745–920)	650 (560–760)	0.001
Genetics**, *n* (%)	26 (68)	26 (68)	N/A	N/A
Patients with no identified mutations	13 (50)	13 (50)		
Patients with identified mutated genes	13 (50)	13 (50)		
• TNFRSF13B	5	5		
• CTLA-4	3	3		
• CXCR4	1	1		
• JAK2	1	1		
• TTC37	1	1		
• CD19	1	1		
• ERCC6l2 heterozygosis	1	1		
• CASP10,	2	2		
• TNFRSF12, 1A	1	1		
• TERT	1	1		
FEV1 (% predicted),edian (IQR)	88.5 (72–107.5)	88 (69.5–106)	99.5 (84–114.2)	0.024
FVC (% predicted), median (IQR)	92 (76–110)	89 (70.5–105.5)	98 (89–114)	0.038
TLC (% predicted), median (IQR)	90 (90–105)	88.5 (75–101)	113 (111–121)	0.009
DLCO (% predicted), median (IQR)	68 (52–80.5)	63 (52–81)	73 (64–79)	ns

*From the diagnosis of CVID to the CT

**Next Generation Sequencing for the coding region and intron-exon junctions of all the genes included in the most updated “International Union of Immunological Societies” genes classification for inborn errors of immunity, available at the time of blood sampling

Legend: *CD21low*, CD21 low B cells; *CT*, computed tomography; *CVID*, Common Variable Immunodeficiency; *DLCO*, carbon monoxide diffusing lung capacity; *FEV1*, Forced Expiratory Volume in the first second; *FVC*, Forced Vital Capacity; *GL-ILD*, Granulomatous and Lymphocytic Interstitial Lung Disease; *IQR*, interquartile range; *ITP*, Immune thrombocytopenic purpura; *MZ*, Marginal Zone B cells; *N/A*, not applicable; *ns*, not significative; *SmB*, Switched memory B cells; *IgG TL*, IgG trough level; *TLC*, Total Lung Capacity

During follow-up, 5 patients developed a lymphoma, 3 of them died, 1 of which for lymphoma progression.

### CT Findings

All CT findings are shown in Table 2. In GL-ILD patients, common lung CT abnormalities were small nodules (37 patients, 97%), scars/bands (35, 92%), consolidations (29, 76%), GGO (27, 71%) and bronchiectasis (27, 71%). Reticulations were present in almost half of patients (18, 47%). Bronchial wall thickening (10, 26%), signs of fibrotic ILD (13, 34%), large nodules (7, 18%), tree in bud (9, 24%), mosaic perfusion (7, 18%), mucous plugs (5, 13%) were less usual. Areas of cavitation/necrosis were never observed. Bronchiectasis were most commonly of a low severity and their prevalence was not significantly different between young and elderly (>65 years) subjects. Small nodules were usually multiple, both solid and ground glass, non-perilymphatic. Considering the whole lung, non-perylymphtic nodules were significantly more frequent than perylimphatic (*p*< 0.0001). The halo sign was observed in 17/37 patients, while calcifications were present in a few subjects. Fibrotic ILD was present in 13 patients. The whole disease was lower field predominant in 35 (92%) patients and diffuse in 3 patients. Irrespective of their frequency, all the investigated findings were usually more frequent in the lower fields as compared to the upper fields. Moreover, GGO, reticulations, fibrotic ILD and tree in bud were more commonly observed in the lower fields only. None of these alterations was more frequent in upper fields only (Supplementary Table 1). The GL-ILD radiological items detected, at significant higher frequency, in the lower fields were fibrosis (*p* = 0.0006), ground glass opacities (*p* = 0.0055), bronchiectasis (*p* = 0.0110), reticulations (*p* = 0.0116) and scars/bands (*p* = 0.0216).

**Table 2:** Patients with airways (a), parenchymal (b) alterations and ancillary findings (c)

	GL-ILD				NON GL-ILD		
	Total	Upper fields	Lower fields	p-value	Total	Upper fields	Lower fields
a) Airways							
Bronchiectasis, *n* (%)	27 (71%)	14 (37%)	26 (68%)	0.0110	14 (37%)	6 (16%)	12 (32%)
Severity							
• Mild	16	7	15		6	3	5
• Moderate	5	3	6		4	2	4
• High	6	4	5		4	1	3
Distribution							
• Central	8	7	4		1	1	1
• Peripheral	9	3	8		11	4	9
• Mixed	10	4	14		2	1	2
BWT, *n* (%)	10 (26%)	6 (16%)	8 (21%)	ns	9 (24%)	7 (19%)	9 (24%)
Mucous Plugs, *n* (%)	5 (13%)	2 (5%)	4 (11%)	ns	11 (29%)	2 (5%)	11 (29%)
• ≤3	3	1	2		10	1	10
• >3	2	1	2		1	1	1
Tree in bud, *n* (%)	9 (24%)	2 (5%)	8 (21%)	ns	6 (16%)	4 (11%)	3 (8%)
Mosaic perfusion, *n* (%)	7 (18%)	6 (16%)	7 (18%)	ns	2 (5%)	0	2 (5%)
b) PARENCHYMA							
Small nodules, *n* (%)	37 (97%)	33 (87%)	37 (97%)	ns	29 (76%)	18 (47%)	27 (71%)
Number							
• ≤3	4	7	7		18	9	9
• >3	33	26	30		11	9	18
Distribution							
• Perilymphatic	4	2	4		3	4	5
• Centrilobular	12	17	10		12	12	13
• Random	21	14	23		14	2	9
Density							
• Solid	2	11	12		17	13	15
• GGO	8	1	2		1	5	1
• Both	27	21	23		11	0	11
Halo sign	17	12	13		6	4	4
Calcification	8	5	4		9	5	5
Large nodules, *n* (%)	7 (18%)	2 (5%)	6 (16%)	ns	2 (5%)	0 (0%)	2 (5%)
Number							
• ≤3	4	2	3		2	0	2
• >3	3	0	3		0	0	0
Density							
• Solid	3	0	4		1	0	1
• GGO	1	2	0		1	0	1
• Both	3	0	2		0	0	0
Halo sign	3	0	3		0	0	0
Shape							
• Rounded/Oval	6	2	5		1	0	1
• Lobulated	1	0	1		1	0	1
• both	0	0	0		0	0	0
Margins							
• Sharp	4	2	3		2	0	2
• Irregular/ill defined	3	0	3		0	0	0
• Mixed	0	0	0		0	0	0
Calcification	0	0	0		0	0	0
Consolidation, *n* (%)	29 (76%)	20 (53%)	29 (76%)	ns	7 (19%)	1 (3%)	8 (21%)
GGO, *n* (%)	27 (71%)	12 (74%)	25 (66%)	0.0055	9 (24%)	4 (11%)	9 (24%)
Reticulations, *n* (%)	18 (47%)	6 (16%)	17 (45%)	0.0116	3 (8%)	2 (5%)	3 (8%)
Fibrotic ILD, *n* (%)	13 (34%)	1 (3%)	13 (34%)	0.0006	1 (3%)	1 (3%)	1 (3%)
Cavitation/Necrosis, *n* (%)	0 (0%)	0 (0%)	0 (0%)	ns	0 (0%)	0 (0%)	0 (0%)
Scars/Bands, *n* (%)	35 (92%)	23 (60%)	34 (89%)	0.0216	27 (71%)	5 (3%)	15 (39%)
c) Other findings							
Pleural effusion, *n* (%)	0 (0%)				0 (0%)		
Enlarged Lymphnodes, *n* (%)	27 (71%)				4 (11%)		
• calcifications	0				0		
Pericardial Effusion, *n* (%)	1 (3%)				1 (3%)		

Numbers in bold: more than 50% of patients

Legend: *GL*=Granulomatous and Lymphocytic; *ILD*=Interstitial Lung Disease; *ns*=not significative; *BWT*=Bronchial Wall Thickening; *GGO*=Ground Glass Opacities

Finally, most subjects had enlarged mediastinal lymph nodes (27, 71%), all without inner calcifications. Pericardial effusion was observed in 2 patients, pleural effusion was never described. Smokers (former or active) did not show any relevant radiological difference as compared to never smokers.

The comparison of GL-ILD CT scans with age- and sex-matched CVID patients without GL-ILD revealed that CT features most significantly associated with GL-ILD (*p*<0.001) were multiple small nodules, consolidations, GGO, reticulations and fibrotic ILD, considering the whole lungs, as well as the presence of enlarged mediastinal lymph nodes. All the statistically significant data are reported in Table 3.

**Table 3:** Frequency of CT findings in GLILD and non-GLILD cohorts. Only significant different CT features are reported

CT Findings	GL-ILD	NON GL-ILD	*p*-value*	Odds ratio	95% CI
Upper fields					
Small nodules (%)	86.84%	48.65%	0.0005	6.967	2.186 to 19.02
• >3 (%)	68.42%	24.32%	0.0002	6.741	2.512 to 19.33
Consolidation (%)	52.63%	2.70%	<0.0001	40	6.566 to 425.9
GGO (%)	31.58%	10.81%	0.0467	3.808	1.133 to 11.59
Lower fields					
Bronchiectasis (%)	68.42%	32.43%	0.0026	4.514	1.613 to 11.26
Mucus plugs (%)	10.53%	29.73%	0.0467	0.2781	0.09055 to 0.9676
Small nodules (%)	97.37%	72.97%	0.0031	13.7	1.995 to 152.4
• >3 (%)	78.95%	48.65%	0.0083	3.958	1.472 to 11.50
Consolidations (%)	76.32%	21.62%	<0.0001	11.68	3.947 to 32.23
GGO (%)	34.21%	2.70%	0.0006	18.72	2.942 to 204.3
RET (%)	65.79%	24.32%	0.0005	5.983	2.260 to 16.73
Fibrotic ILD (%)	44.74%	8.11%	0.0005	9.175	2.568 to 31.46
Scars/ bands (%)	84.21%	56.76%	0.0116	4.063	1.342 to 12.49
Whole lungs					
Bronchiectasis (%)	71.05%	37.84%	0.0054	4.032	1.555 to 9.952
Small nodules (%)	97.37%	78.38%	0.014	10.21	1.338 to 115.9
• >3 (%)	86.84%	48.65%	0.0005	6.967	2.186 to 19.02
Consolidations (%)	76.32%	21.62%	<0.0001	11.68	3.947 to 32.23
GGO (%)	71.05 %	24.32%	<0.0001	7.636	2.798 to 21.30
RET (%)	47.37%	8.11%	0.0002	10.2	2.798 to 21.30
Fibrotic ILD (%)	34.21%	2.70%	0.0006	18.72	2.942 to 204.3
Scars/bands (%)	92.11%	64.86%	0.0049	6.319	1.672 to 22.08
Mediastinum					
Enlarged Lymphnodes (%)	71.05%	10.81%	<0.0001	20.25	5.489 to 59.99

*Comparisons made by Fisher’s exact test

Legend: *CI*, confidence interval; *CT*, computed tomography; *GL*, granulomatous and lymphocytic; *ILD*, interstitial lung disease; *GGO*, ground glass opacities

### Correlation with PFT and Immunological Data in GL-ILD Patients

Having determined a significant predominance of disease burden in the lower lung fields, we searched for potential association between such aforementioned, differentially distributed, radiological findings and the clinical-immunological data.

In the presence of fibrotic ILD, patients had a poorer lung function, as expressed by reduced Forced Expiratory Volume in the first second, % predicted (FEV1%) (*p *= 0.0346), Forced Vital Capacity, % predicted (FVC%) (*p *= 0.0271), Total Lung Capacity, % predicted (TLC%) (*p *= 0.0438) and carbon monoxide diffusing lung capacity, % predicted (DLCO%) (*p*=0.0030), lower IgA (*p *= 0.0108) and IgM (*p *= 0.0249) at diagnosis, and a prevalence in female gender (*p *= 0.0773).

Similarly, patients with GGO, compared to those without, showed lower IgA levels at diagnosis (*p *= 0.0055), reduced TLC% predicted (*p *= 0.0003), lower peripheral blood leukocytes count (*p *= 0.0125) and higher CD4+ T cells percentage (*p *= 0.0334).

Lower IgA and IgG at diagnosis (*p *= 0.0039 and *p *= 0.0064, respectively) and worse pulmonary flows and volumes, expressed as lower FEV1% (*p* = 0.0239), were found also in patients with bronchiectasis.

Finally, the presence of reticulation on CT scan was more frequent in patients with decreased lung performance, expressed by dropping in FEV1% (*p *= 0.0059), FVC% (*p *= 0.0057), TLC% (*p *= 0.0004) and DLCO% (*p *= 0.0030) predicted, while patients with reduced lymphocyte counts (*p *= 0.0059) showed a higher occurrence of scars/bands.

Interestingly, patients with enlarged mediastinal lymph nodes had higher peripheral plasmablasts (*p *= 0.0018).

Considering the presence of granuloma on biopsy, we did not detect any significant difference in radiological appearance nor in immunological or lung function parameters, as compared to patients without histological evidence of granuloma.

Results of the univariate logistic regression analyses for each of those CT items are shown in Supplementary Table 2.

### Possible Determinants of GL-ILD Treatment

Considering GL-ILD treatment as criteria to subdivide our cohort, we did not identify any CT finding differentially distributed between treated and untreated patients (only the presence of lower field consolidations had a *p*-value of approaching significance, *p*=0.0577), nor any baseline lung function parameter. On the contrary, GL-ILD-treated patients presented lower MZ B cells percentage (*p*=0.0181) and higher CD21low B cells percentage (*p*=0.0035), whereas low IgA levels (in GL-ILD-treated patients vs untreated) where close to reach statistical significance (*p*=0.0802).

**Fig. 1:**
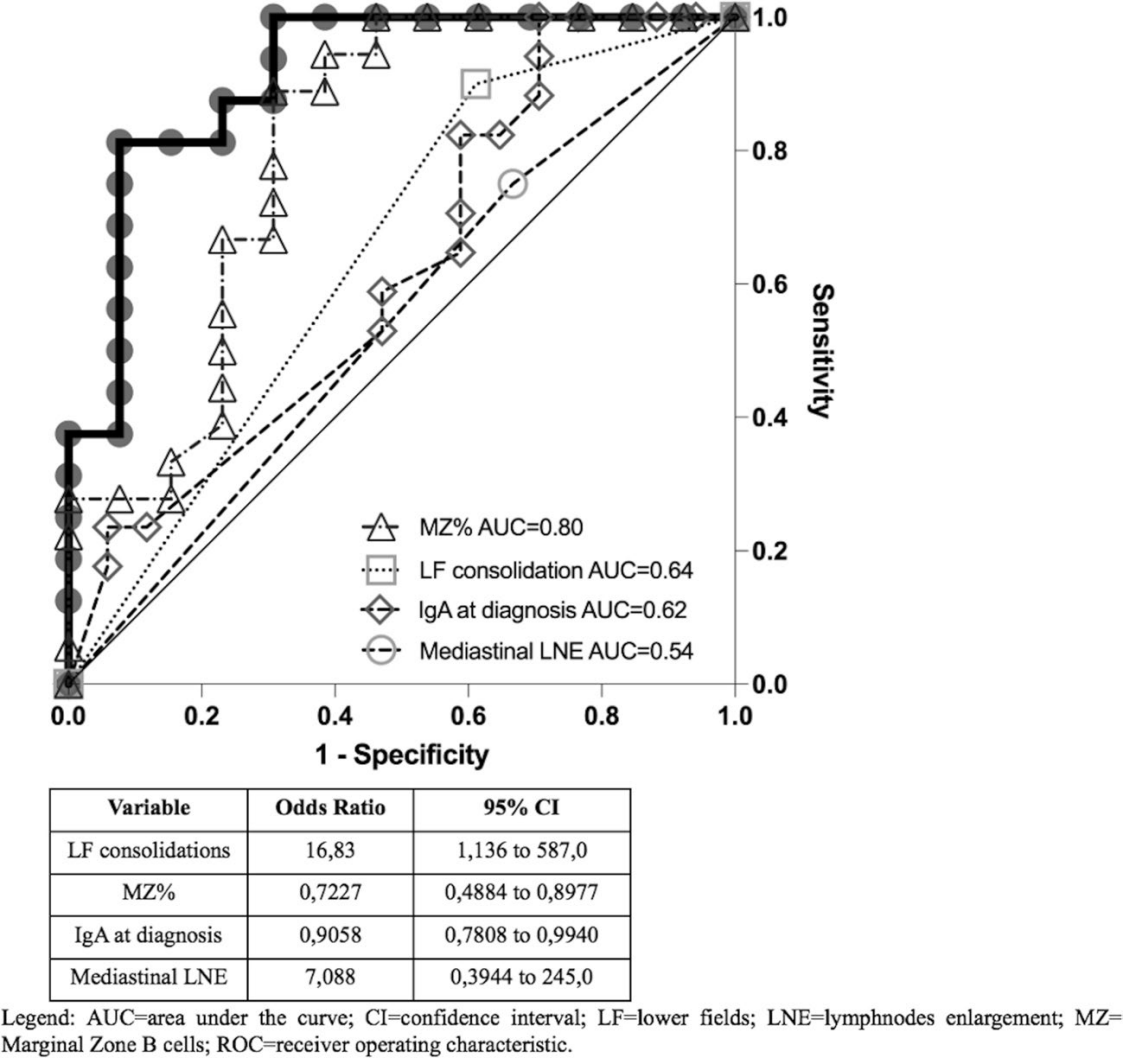
ROC curve of the multiple logistic regression model. The ROC curve of the multiple logistic regression model underlies an AUC of 0.91 (*p*=0.0002). The graph also shows the ROC curves for the logistic regression analysis of the single variables

At univariate logistic regression analysis, CD21low and MZ B cells percentage, IgA levels at diagnosis and the presence of consolidations in the lower fields of CT scan, were found to have the higher likelihood in GL-ILD treatment prediction. The final multivariate logistic regression model including MZ B cells percentage and IgA levels at diagnosis as immunological covariates and the CT evidence of consolidations in the lower fields and mediastinal lymph nodes enlargement, allowed us to reach a better predictive performance for GL-ILD treatment (sensitivity 87.50% and specificity 76.92%, using a cut-off value of 0.5). The joint analysis of these four variables together in a multiple logistic regression model yielded an AUC of 0.91 (95% CI: 0.80–1.0) (Figure 1).

## Discussion

**Fig. 2:**
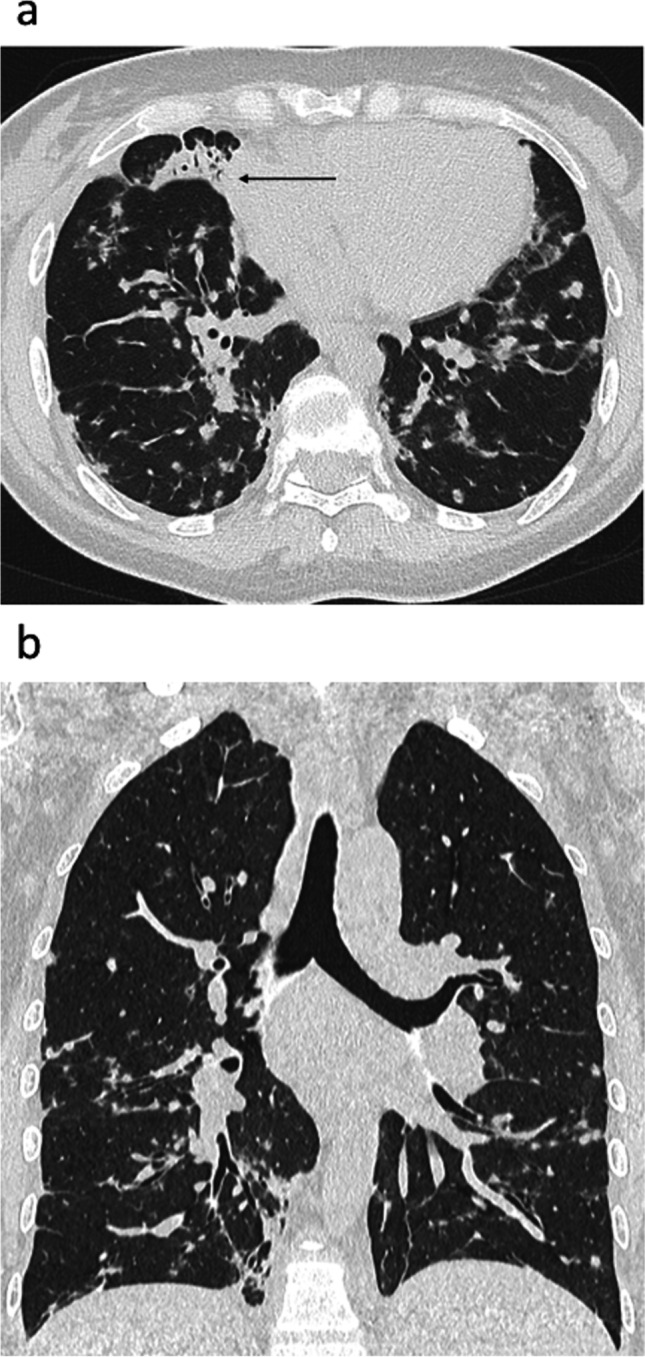
Axial (**a**) and Coronal (**b**) reconstructions. Small nodules with a random distribution. Some of them show a ground glass halo (halo sign). A consolidation is also present in the medium lobe (**a**, black arrow). The disease demonstrates a lower field predominance (**b**)

**Fig. 3:**
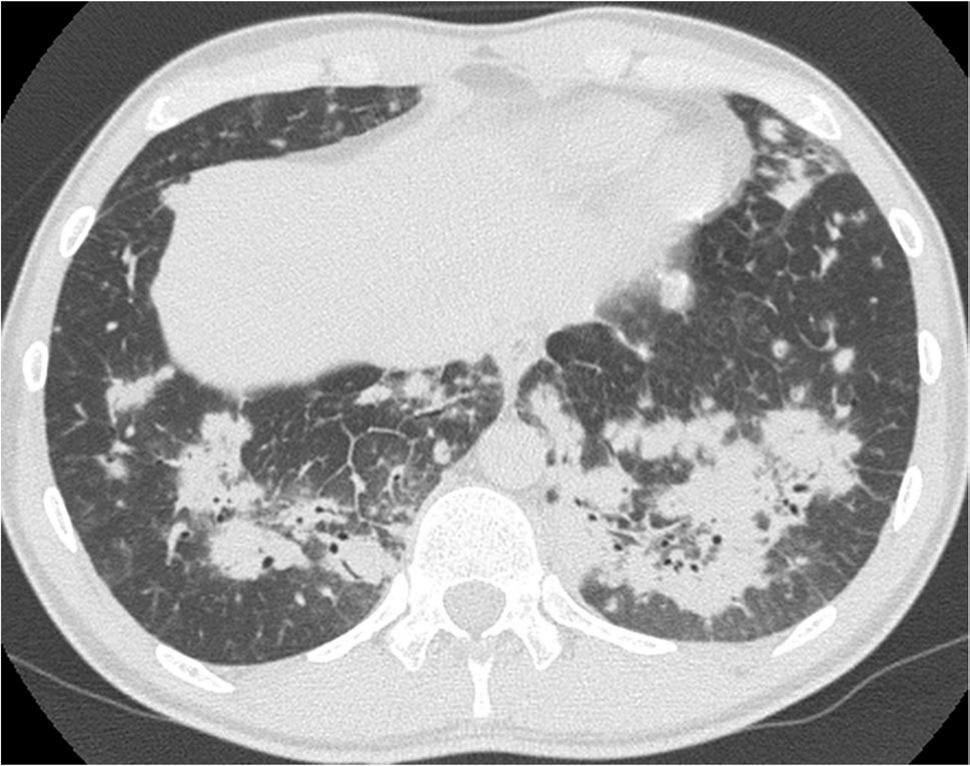
Lower field, areas of peribronchial confluent consolidation. Nodules, areas of Ground Glass Opacities and reticulations are also present

**Fig. 4:**
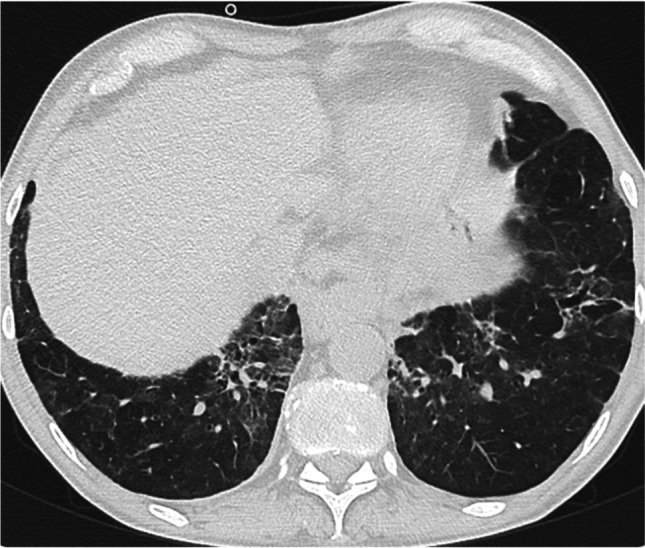
Signs of fibrotic ILD in the lower field: traction bronchiectasis and parenchymal distortions inside areas of Ground Glass Opacities. Areas of Ground Glass opacities without fibrosis are also present

In this study, the most common alterations in chest CT scan of GL-ILD patients (observed in more than 50% of cases) were bronchiectasis (commonly central and of low severity), small nodules (without a perilymphatic distribution) (Figure 2), consolidations (Figure 3), GGO, parenchymal scars/bands and enlarged mediastinal lymph nodes. At variance, previous works reported consolidations and GGO as less usual findings [13, 28]. In our opinion, this difference could be related to different phases of the disease and/or enrollment criteria for CT evaluation: in our study, the low frequency of fibrotic ILD (Figure 4) compared to consolidation and GGO, suggests that these latter were usually sustained by early-inflammatory disease, while features of fibrotic ILD may be expression of an end-stage ILD. When comparing with CVID patients without GL-ILD, we found that small nodules (especially if >3), GGO, consolidations, reticulations, fibrotic ILD and lymph nodes enlargement should raise the suspicion of GL-ILD with more confidence, matching what was previously observed by Cinetto et al. [9].

GL-ILD was usually predominant at lower fields (Figure 2), confirming that lung bases are the most involved parts, as firstly observed by Torigian et al. [21] and confirmed by Pac et al. [20] in a small cohort of subjects with primary immunodeficiency, mostly CVID. Moreover, considering the chronic evolutions of ILDs, the observation that reticulations and fibrotic ILD were significantly more frequent in lower fields raises the hypothesis that lung bases are firstly involved in a temporal perspective.

Another interesting finding regards the distribution of nodules. Radiologically, nodules centered in the secondary lobule without contact with pleural, peri-bronchovascular surfaces or interlobular septa may be described as centrilobular, usually sustained by a bronchiolar spread of inflammation. Conversely, nodules that are predominantly located along pleural, peri-bronchovascular surfaces or interlobular septa may be described as peri-lymphatic. Lastly, if they are distributed without any predominance, a random pattern is defined, usually due to hematogenous metastatic or infective spread [29]. In our study, nodules were usually non-perylymphatic. This result was partially unexpected, since a perilymphatic distribution is the hallmark of lung diseases involving the lymphatic structures and data in literature, derived from small cohorts, are conflicting [20, 21, 30]. However, in our opinion, in CVID patients, small inhaled antigens could determine a peribronchiolar reaction with an increase in centrilobular nodules, justifying the relative lower frequency of perilymphtic nodules.

**Fig. 5:**
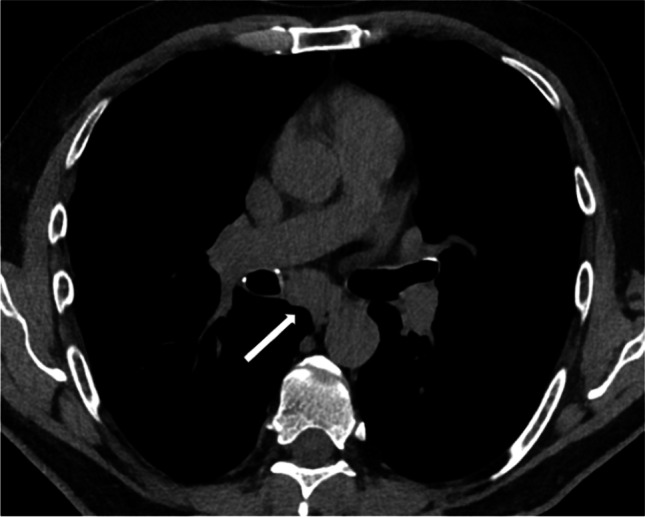
Enlarged subcarinal lymph node (white arrow) without calcifications

**Fig. 6:**
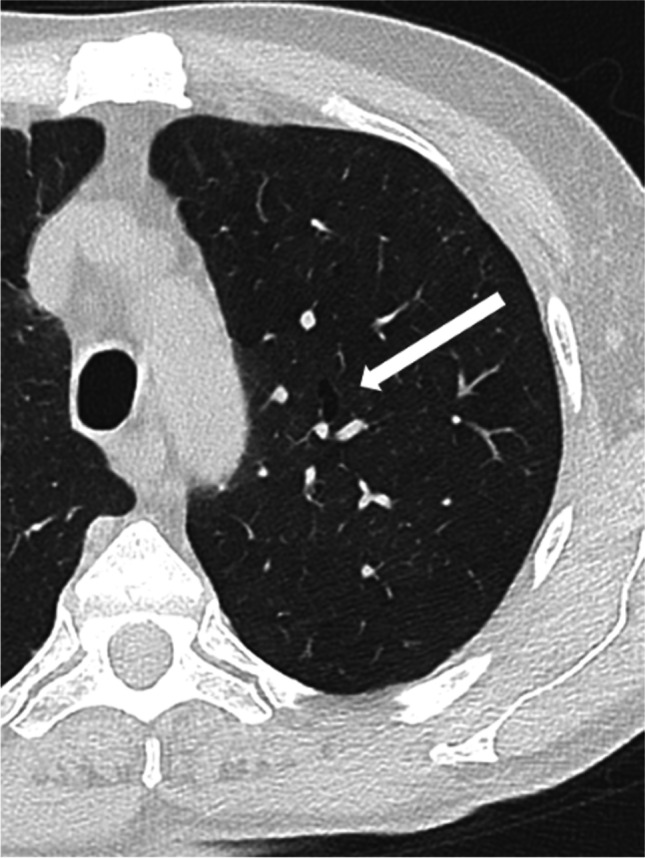
A low grade bronchiectasis without sign of GL-ILD in the left upper field (white arrow)

Therefore, a predominant lower field involvement and a predominant non-perilymphatic nodules distribution may be helpful in differential diagnosis, in particular with sarcoidosis [5, 18, 30]. In fact, the typical radiologic features of pulmonary sarcoidosis are perylimphatic nodules involving middle-upper fields [18, 30]. Moreover, in sarcoidosis, bronchiectasis is part of the fibrotic stage, actually representing traction bronchiectasis inside fibrosis [18]. In our patients, fibrotic ILD was a way less common than bronchiectasis; this suggests that, in GL-ILD, bronchiectasis may be observed as a “pure” finding (Figure 6) rather than secondary to fibrosis. In line with this hypothesis, we did not find a relationship between bronchiectasis and DLCO reduction [31]. Non-traction bronchiectasis may thus be not a specific feature of GL-ILD, but the result of chronic airway damage [1]. Lastly, although enlarged lymph nodes may be observed in both affections (Figure 5), we found no mediastinal lymph nodes calcifications, a finding that might instead suggest sarcoidosis [18]. Other potential concerns for differential diagnosis are represented by consolidations, tree in bud appearance, bronchial wall thickening and mucous plugs, all possible signs of infections that may also be present in GL-ILD. Similarly, we often observed a halo sign surrounding solid lung nodules that can be a manifestation of both organizing pneumonia and different infections, e.g. opportunistic infection, as invasive aspergillosis [23]. However, we did not observe cavitation/necrosis that could instead support the hypothesis of a superimposed infective pneumonia [1, 19]. Another challenging differential diagnosis may be represented by lung lymphomas, in this at-risk population. Unfortunately, lymphomas have a polymorphic appearance [17] and we are not aware of any feature for a confident CT differential diagnosis.

In terms of correlation between radiologic findings and lung function, we also observed that reticulations and fibrotic ILD were associated with a reduction of FEV1%, FVC%, TLC% and DLCO% predicted, as observed in pulmonary fibrosis [31, 32]; the high proportion of patients with reticulation that had also features of fibrotic ILD could justify the results. Alternatively, reticulations might also be the expression of tiny fibrosis in the absence of traction bronchiectasis and parenchymal distortions. Moreover, we identified some peculiar immunological features related to the CT abnormalities. As already reported, low IgA and IgG are associated with GL-ILD diagnosis [9], and low IgA are described also in CVID patients with bronchiectasis [3, 33]. Thus, the correlation between some radiological items (fibrosis, GGO and bronchiectasis) and low immunoglobulin levels in our GL-ILD cohort was not surprising. On the contrary, the association of low white blood cells count and higher CD4+ T cells in peripheral blood with GGO may reflect a GL-ILD-related perturbation in leukocytes trafficking, with preferential accumulation of CD4+ T cells in the lungs airways [34] and parenchima [35], and a possible expansion of memory CD4+ T cells differentiated towards a CXCR3+CCR6− Th1 phenotype [36].

We then focused on identifying the relevant factors, available at the time of the first GL-ILD radiological suspicion, possibly related with the likelihood of subsequent GL-ILD specific treatment. In literature, we found no similar approaches regarding this specific topic, as the available studies focus mainly on longitudinal evaluation of pulmonary function tests prior to GL-ILD treatment, in order to define progressive disease. A recent study [37] also reported the performance of a CT scoring system in predicting progressive *vs* stable disease, which is, in our knowledge, the only attempt made to identify radiological elements related to GL-ILD worsening. At present, even though no guidelines provide specific indication for the beginning of GL-ILD immunosuppressive treatment, it is accepted that immunosuppression is required in patients with deteriorating pulmonary function, and/or with relevant symptoms and/or with worsening radiological abnormalities [38]. We thus considered our CT findings, clinical and immunological parameters, finally identifying a small number of immunological and radiological variables functional to obtain a better model performance to possibly foresee the need for a specific GL-ILD therapy, even before evidence of radiological progression or decline of lung function parameters. As radiological parameters, we selected the presence of parenchymal consolidations that was superior to any baseline lung function parameter in increasing the model performance for GL-ILD treatment prediction, and mediastinal lymph nodes enlargement. These two findings may provide not only evidence of active GL-ILD, but are also useful and easy-to-detect elements for evaluating treatment response [38]. In terms of immunological variables, we included, in our model, serum IgA levels at CVID diagnosis and circulating MZ B cells at the time of the CT scan. MZ B cells are functionally considered a first line defense, especially against encapsulated bacteria, and a housekeeping B cellular subset, because of self-antigen and foreign-antigen clearance activity (through their poly-reactive B cell receptors) and secretion of natural IgM antibodies. Given the intrinsic auto reactivity and high sensitivity to stimulation through Toll-like receptors, MZ B cells compartment is tightly regulated, and currently available evidence suggests that it may be implicated in human autoimmunity [39]. Furthermore, extra splenic MZ B cells can localize in tertiary lymphoid structures, including ectopic germinal centers that are often formed in target tissues of autoimmune processes [40] and, interestingly, have also been described in GL-ILD[35]. In this environment, MZ B cells can favor antigen delivery to ectopic germinal centers [41] and/or directly present antigens to T cells [41]. Such peculiar migration may explain our finding of reduced circulating MZ B cells in patients who subsequently needed GL-ILD treatment. On the contrary, CD21low B cells have been found to be expanded in CVID patients with immune dysregulation (and to correlate with GL-ILD diagnosis), probably because of the strong T cell-derived IFN-gamma environment in blood and secondary lymphoid organs [42]. Accordingly, in our study, radiological signs of active lung inflammation (like GGO) and GL-ILD treatment itself correlated with increased CD4+ T cells percentage and CD21 low B cells expansion, respectively. However, we decided not to include CD21low expansion in the multivariate model for predicting the GL-ILD treatment in order to minimize the number of variables entered and because its elimination resulted in a minimal loss of the explained variance. These results need further confirmation; however, in addition to the already known relevance of CD21low, MZ B cells could in the future gain importance if studied ad hoc to verify their prognostic impact on the need for treatment.

Lung function parameters were not found helpful in improving the model performance; this is possibly due to the single measurement at the time of CT scan, with no possibility to evaluate decline, and/or to the potentially earlier evaluation compared to other studies.

Our study presents some limitations. Firstly, being a retrospective study, laboratory and pulmonary function tests, as well as CT parameters, were performed in different centers without a previous definition of shared protocols/settings. Then, most of the CT alterations were not assessed with a quantitative or semiquantitative score that may be useful to better define, for instance, the prevalence of alterations between upper and lower fields. Moreover, to confirm the usefulness of the provided elements for a differential diagnosis, a comparison with patients with the other aforementioned diseases is needed. Finally, because this study was not designed to specifically investigate the impact of the different B cell subpopulations on the physiopathology of GL-ILD, further studies are needed to specifically address these findings.

## Conclusions

In conclusion, as resulted from our cohort, the most common CT findings in GL-ILD, before treatment, are small nodules with a non-perilymphatic distribution, consolidations, GGO, scars/bands and bronchiectasis. Reticulations may also be observed. Less common features are bronchial wall thickening, signs of fibrotic ILD, large nodules, tree in bud, mosaic perfusion and mucous plugs. GL-ILD is usually prevalent in lower fields. Multiple small nodules, GGO, consolidations, reticulations, fibrotic ILD and lymph nodes enlargement may raise the suspicion of GL-ILD in CVID patients.

A model composed by MZ B cells percentage, IgA at diagnosis, lower field consolidations at CT and mediastinal lymph nodes enlargement may be predictive of the need for a specific GL-ILD therapy.

### Supplementary information

ESM 1 (DOCX 18 kb)

## Data Availability

Data are available upon request. For further information, please contact: cinzia.milito@uniroma1.it
