# Supplementary material for: Common and Uncommon CT Findings in CVID-Related GL-ILD: Correlations with Clinical Parameters, Therapeutic Decisions and Potential Implications in the Differential Diagnosis
Source: J Clin Immunol. 2023 Aug 7;43(8):1903–15. doi: 10.1007/s10875-023-01552-1 (PMC10661728; doi:10.1007/s10875-023-01552-1)
Supplement: Supplementary file 1 — (DOCX 18 kb) [file 10875_2023_1552_MOESM1_ESM.docx]

**Supplementary Table 1.** Patients with airways and parenchymal alterations, observed in both fields or in one field only.

| **CT FINDINGS** | **TOTAL** | **BOTH FIELDS**  **n (%)** | **UPPER FIELDS ONLY n (%)** | **LOWER FIELDS ONLY n (%)** |
| --- | --- | --- | --- | --- |
| **Bronchiectasis** | 27 | **13 (48)** | 1 (4) | **13 (48)** |
| **Bronchial Wall Thickening** | 10 | **4 (40)** | 2 (20) | **4 (40)** |
| **Mucous Plugs** | 5 | 1 (20) | 1 (20) | 3 (60) |
| **Mosaic perfusion** | 7 | **6 (86)** | 0 (0) | 1 (14) |
| **Tree in bud** | 9 | 1 (11) | 1(11) | **7 (78)** |
| **Small nodules** | 37 | **33 (89)** | 0 | 4 (11) |
| **Large nodules** | 7 | 1 (14) | 1 (14) | **5 (72)** |
| **Consolidation** | 29 | **20 (69)** | 0 (0) | 9 (31) |
| **Ground Glass Opacities** | 27 | 10 (37) | 2 (7) | **15 (56)** |
| **Reticulations** | 18 | 5 (28) | 1 (4) | **12 (68)** |
| **Fibrotic ILD** | 13 | 1 (8) | 0 (0) | **12 (92)** |
| **Cavitation/necrosis** | 0 | 0 (0) | 0 (0) | 0 (0) |
| **Bands** | 35 | **19 (54)** | 3 (9) | 13 (37) |

Numbers in bold: field with the highest frequency of the specific CT finding

Legend: ILD= Interstitial Lung Disease

**Supplementary Table 2.** Univariate logistic regression analysis for different possible GLILD radiological items predictors and GLILD treatment predictors. Only the relevant findings are reported below.

|  | **Odds Ratio** | **95% C.I.** | **p value** | **AUC** |
| --- | --- | --- | --- | --- |
| **GL-ILD treatment** |  |  |  |  |
| IgA at diagnosis (mg/dl) | 0,9572 | 0,9010 to 0,9983 | 0,0404 | 0,6194 |
| CD21low B cells (%) | 1,088 | 1,025 to 1,187 | 0,003 | 0,7758 |
| MZ B cells (%) | 0,8235 | 0,6796 to 0,9306 | 0,0003 | 0,8013 |
| LF consolidations | 5,727 | 1,143 to 43,35 | 0,0331 | 0,6444 |
| Mediastinal lymph nodes enlargement | 1,500 | 0,3652 to 6,398 | 0,5718 | 0,5417 |
| **LF Fibrosis** |  |  |  |  |
| Male gender | 0,197 | 0,02677 to 0,9299 | 0,0396 | 0,6631 |
| IgA at diagnosis (mg/dl) | 0,9128 | 0,7968 to 0,9847 | 0,0101 | 0,7411 |
| IgM at diagnosis (mg/dl) | 0,9605 | 0,8922 to 0,9983 | 0,0193 | 0,7115 |
| FEV1 (% predicted) | 0,9645 | 0,9271 to 0,9966 | 0,0293 | 0,7062 |
| FVC (% predicted) | 0,961 | 0,9220 to 0,9949 | 0,0235 | 0,7077 |
| TLC (% predicted) | 0,9376 | 0,8761 to 0,9873 | 0,0124 | 0,7682 |
| DLCO (% predicted) | 0,9188 | 0,8463 to 0,9729 | 0,0018 | 0,8113 |
| **LF Ground Glass Opacities** |  |  |  |  |
| WBCs (cells/uL) | 0,9997 | 0,9994 to 0,9999 | 0,0125 | 0,6708 |
| Ly (cells/uL) | 0,9991 | 0,9979 to 0,9999 | 0,0282 | 0,7118 |
| CD4 T cells (%) | 1,09 | 1,005 to 1,187 | 0,0358 | 0,7326 |
| IgA at diagnosis (mg/dl) | 0,9455 | 0,8903 to 0,9874 | 0,0084 | 0,7803 |
| TLC (% predicted) | 0,8461 | 0,7121 to 0,9359 | <0,0001 | 0,9063 |
| **LF Bronchiectasis** |  |  |  |  |
| IgG at diagnosis (mg/dl) | 0,9938 | 0,9882 to 0,9983 | 0,0061 | 0,7468 |
| IgA at diagnosis (mg/dl) | 0,9501 | 0,8994 to 0,9898 | 0,0122 | 0,7938 |
| FEV1 (% predicted) | 0,9601 | 0,9198 to 0,9938 | 0,0192 | 0,734 |
| FVC (% predicted) | 0,9644 | 0,9249 to 0,9989 | 0,0427 | 0,6923 |
| TLC (% predicted) | 0,9483 | 0,8870 to 0,9996 | 0,0481 | 0,7339 |
| **LF Reticulations** |  |  |  |  |
| CD21low B cells (%) | 1,045 | 1,001 to 1,104 | 0,0469 | 0,6528 |
| FEV1 (% predicted) | 0,9534 | 0,9135 to 0,9865 | 0,0046 | 0,7633 |
| FVC (% predicted) | 0,9519 | 0,9114 to 0,9861 | 0,0048 | 0,7549 |
| TLC (% predicted) | 0,888 | 0,7976 to 0,9544 | 0,0002 | 0,8672 |
| DLCO (% predicted) | 0,9229 | 0,8563 to 0,9744 | 0,0021 | 0,7857 |
| **LF Scars/distelectatic bands** |  |  |  |  |
| Ly (cells/uL) | 0,999 | 0,9975 to 0,9998 | 0,0118 | 0,8 |

Legend: CD21low=CD21 low B cells; DLCO=carbon monoxide diffusing lung capacity; FEV1=Forced Expiratory Volume in the first second; FVC=Forced Vital Capacity; GL-ILD=Granulomatous and Lymphocytic Interstitial Lung Disease; IQR=interquartile range; ITP=Immune thrombocytopenic purpura; LF =lower fields; Ly=lymphocytes; MZ= Marginal Zone B cells; TLC=Total Lung Capacity; WBCs=white blood cells.
